# Abstracts from the Fourth Bioelectronic Medicine Summit: Technology Targeting Molecular Mechanisms: BEM, Virtual, 23-24 September 2020

**DOI:** 10.1186/s42234-020-00060-6

**Published:** 2020-12-23

**Hosted virtually by the Feinstein Institutes for Medical Research, September 23, 24th, 2020**

**Sponsored by CorTec (Freiburg, Germany), BMC-Bioelectronic Medicine (journal), and Feinstein Institutes for Medical Research/Northwell Health**

## P1. Factors affecting thresholds for acute and long-term vagus nerve stimulation

### Umair Ahmed, MD, Maria Lopez, BS, Yao-Chuan Chang, PhD, Loren Rieth, PhD^,^ Timir D. Chaudhury, PhD, Yousef Al-Abed, PhD, and Stavros Zanos, MD PhD

#### Institute of Bioelectronic Medicine, Feinstein Institutes for Medical Research, Manhasset, New York, US

##### **Correspondence:** Stavros Zanos

In animal experiments involving vagus nerve stimulation (VNS), standardization of dose within and across subjects is achieved by determining the physiological threshold (PT), often defined as the minimum stimulation intensity needed to elicit a physiological response. Factors affecting PT in the context of VNS in rodents, the most common preclinical animal model, have not been studied systematically. We determined the effect on PT of common factors known to play in role in neurostimulation. Adult rats were acutely or chronically implanted with a vagus nerve electrode. Acute experiments were performed under anesthesia while experiments involving chronic implants were performed either under anesthetized or in awake conditions. Physiological responses to VNS of increasing intensity included changes in blood pressure, bradycardia and apnea, in anesthetized and awake experiments. PT was smaller when the VN electrode was better insulated. PT and in vivo impedance increased with implant age over the course of 4-6 weeks, but PT had a weak correlation with impedance on a single measurement basis. PT was lowest in awake condition, followed by anesthesia with ketamine/xylazine and with isoflurane. Similarly, the cardioinhibitory effects of VNS for supra-threshold stimulation intensities were maximum in awake condition, followed by ketamine/xylazine, and lastly isoflurane.

## P2. Electrically-evoked vagus nerve recordings using transmural endoscopy in a swine model

### Nikunj Bhagat, PhD^1^, Anil Vegesna, MD^1^, Chunyan Li, PhD^1,2^, Loren Rieth, PhD^1,2^, Richard Ramdeo^1^, Chad Bouton, MSc^1,2^, Horacio Rilo, MD^2,3^, and Larry Miller, MD^1,3^

#### ^1^Institute of Bioelectronic Medicine, Feinstein Institutes for Medical Research, Northwell Health, Manhasset, New York, US; ^2^Zucker School of Medicine at Hofstra/Northwell, Hempstead, New York, US; ^3^North Shore University Hospital-Northwell Health, Manhasset, New York, US

##### **Correspondence:** Larry Miller

Transmural endoscopy for the purposes of performing per oral endoscopic myotomy (POEM) is a well-established and routine procedure for treating achalasia. During this procedure, an endoscopic tunnel is formed between the esophageal mucosa to the outer muscle layers, thereby gaining access to thoracic and abdominal cavities. Previously, we have shown that it is possible to identify and localize peripheral branches of the vagus nerve using commercially available endoscopes with digital/optical magnification and narrow band imaging. In this study, we explore the feasibility of recording nerve activity from the esophageal and gastric vagus branches within the abdominal and thoracic cavity in a swine model, by using transmural endoscopy. We will use custom-built nerve cuff and spiral electrodes that can be placed endoscopically to monitor for nerve activity and stimulate at different sites along the peripheral vagal branches. Additionally, we will also record the evoked compound action potentials (CAPs) in response to cervical and auricular vagus nerve stimulation applied directly on the nerve.

Transmural endoscopy opens up the possibility of localizing and recording from the vagal nerve branches via a minimally invasive surgical procedure. Further studies are needed to explore its potential for delivering bioelectronic medicine.

## P3. Transcutaneous spinal cord stimulation selectivity and optimization for hand restoration in tetraplegia

### Nikunj Bhagat, PhD^1^, Santosh Chandrashekaran PhD^1^, Richard Ramdeo^1^, Stephan Bickel, MD, PhD^1,2^, Jose Herrero, PhD^1,2^, Adam Stein, MD^2^, Ashesh Mehta, MD, PhD^1,2^ and Chad Bouton, MSc^1,2^

#### ^1^Institute of Bioelectronic Medicine, Feinstein Institutes for Medical Research, Northwell Health, Manhasset, New York, US; ^2^Zucker School of Medicine at Hofstra/Northwell, Hempstead, New York, US

##### **Correspondence:** Chad Bouton

Spinal cord injury (SCI) affects approximately 18,000 individuals every year in the United States, with over half of the cases resulting in upper limb impairment. Restoring hand movement and dexterity is one of the key, albeit largely unmet, priorities of SCI survivors. Recently, transcutaneous spinal cord stimulation (tSCS) in conjunction with activity-based training has shown considerable promise in improving motor performance of incomplete SCI patients. However, conventional tSCS training requires intensive physical therapy for long periods, typically 80 sessions. This is because conventional tSCS uses an all-or-none stimulation pattern that simultaneously activates multiple motor pools and hence, it requires a needlessly laborious effort to isolate multiple hand movements, which is not clinically viable.

In this study we explore the effect of stimulation pattern and electrode area on targeting specific spinal nerves via tSCS for hand restoration. We have developed a custom transcutaneous neurostimulator with fatigue-resistant waveforms and high-density electrodes (FocalStim) that allow highly focalized activations of nerve fibers. Using FocalStim, we can generate individual or burst mode stimulation patterns, as well as, vary the electrode area from 100mm^2^ to 1000mm^2^. Further, by recording hand movements and muscle activity through electromyogram recordings of both able-bodied and SCI participants during tSCS, we can potentially identify the optimal stimulation parameters and electrode sizes for targeting specific hand movements using tSCS. Future studies will explore automatic optimization of tSCS combined with intent-driven (through an implantable brain interface and non-invasive wearable sensing technologies) targeting of specific spinal nerves and its benefits on hand restoration.

## P4. Highly focal somatosensory percepts at fingertips evoked reliably by SEEG electrodes: an approach for sensory BCI

### Santosh Chandrasekaran^1^, Stephan Bickel^2,3,4^, Jose Herrero^2^, Noah Markowitz^2^, Elizabeth Espinal^2^, Kim Joo-won^5^, Nikunj Bhagat^1^, Richard Ramdeo^1^, Gordon Xu^5^, Matt Glasser^6^, Chad Bouton^1^ and Ashesh Mehta^2,3^

#### ^1^Neural Bypass and Brain Computer Interface Laboratory, Institute of Bioelectronic Medicine, Feinstein Institutes for Medical Research, Northwell Health, Manhasset, NY, USA; ^2^The Human Brain Mapping Laboratory, Feinstein Institutes for Medical Research, Northwell Health, Manhasset, NY, US; ^3^Department of Neurosurgery and ^4^Neurology, Donald and Barbara Zucker School of Medicine at Hofstra/Northwell, Manhasset, NY, US; ^5^Baylor College of Medicine, Houston, TX, US; ^6^Departments of Radiology and Neuroscience, Washington University School of Medicine, St. Louis, MO, US

##### **Correspondence:** Ashesh Mehta

Artificial somatosensory feedback is an important component for a sensorimotor brain-computer interface system that is intuitive to use and reduces dependence on visual feedback. For people with tetraplegia, targeted electrical stimulation of the somatosensory cortex has been used to provide somatotopically relevant sensory feedback. Currently, microelectrode arrays and electrocorticography (ECoG) grids are the most widely used modes of delivering such feedback through intracortical stimulation. These two kinds of electrode arrays are complementary in their advantages and deficiencies. While microelectrode arrays evoke highly focal percepts, they provide access to a very limited area of the cortex whereas ECoG grids provide a wider coverage of the cortex but evoke more diffuse percepts. In addition, neither approach can access the sulcal areas of the somatosensory cortex where the receptive fields pertaining to the fingertips are located in humans. Here, we reliably target the hand regions of the primary somatosensory cortex stereotactic electroencephalography (SEEG) depth electrodes guided by high-resolution fMRI and myelin maps. The SEEG electrodes allow us to access even the sulcal regions S1. We elicited highly focal sensory percepts in the fingers, including fingertips, and the hand. Using within-patient comparisons, we also show that percepts evoked by SEEG electrodes are more focal and can be localized to fingertips more often than those evoked by high density ECoG grid electrodes.

## P5. Electrical and mechanical lifetime of helical leads developed for an intracranial cochlear implant

### Christine Crosfield^1^, Dave Warren, PhD^2^, Moritz Leber^3^, Vinh Ngo^3^, Rohit Sharma^4^, Prashant Tathireddy^4^, Jason Wright^1^, Joanne Peragine^1^, Tao Sun, PhD^1^, Loren Rieth, PhD^1^

#### ^1^Institute of Bioelectronic Medicine, Feinstein Institutes for Medical Research, Manhasset, New York, US; ^2^University of Utah, Salt Lake City, Utah, US; ^3^Blackrock Microsystems, Salt Lake City, Utah, US; ^4^Applied Biosensors, Salt Lake City, Utah, US

##### **Correspondence:** Loren Rieth

Multi-conductor electrical leads are a critical and underappreciated aspect of implanted medical devices, and they represent one of the largest failure mechanisms for marketed devices such as DBS and VNS implants. We are developing a new helical lead with gold wires as part of a large NIH-funded program to develop and translate a new hearing prosthesis that uses a penetrating Utah array in combination with a marketed cochlear stimulator. The helical leads for this project are tested to confirm compliance with requirements for cochlear implants (e.g. AAMI/ANSI CI86:2017), while safe and effective for use in the small clinical investigations. The mechanical tests are to evaluate lead performance for surgical use, minimize tethering forces, enable anchoring, and have sufficient fatigue resistance for our study. The helical leads joining the Auditory Nerve Utah Slanted electrode (AN-USEA) and the MED-EL Synchrony 2 stimulator pass the testing standards with expected biomechanical loads, in which are demonstrated with vigorous benchtop tests following a 10-day saline pre-soak and sterilization including: continuity, leakage current (<0.1 μA), bend testing (>100,000 bend cycles at ±15° and 2 Hz frequency), tensile testing to at least 10% elongation, and insulation test between channels in the wire bundle to have impedance values of >100 kΩ. The helical leads are exposed to representative stresses and have not shown signs of failure visually, electrically, nor mechanically which illustrates the robustness and flexibility of the leads. Consistent with standards for cochlear implants, the helical leads show to be a resilient and reliable component of cochlear devices.

## P6. A method to quantify autonomic nervous system function in healthy, able-bodied individuals

### Shubham Debnath, PhD^1^, Todd J. Levy, MS^1^, Theodoros P. Zanos, PhD^1,2^

#### ^1^Institute of Bioelectronic Medicine, Feinstein Institutes for Medical Research, Northwell Health, Manhasset, New York, US; ^2^Donald and Barbara Zucker School of Medicine at Hofstra/Northwell, Hempstead, New York, US

##### **Correspondence:** Theodoros P. Zanos

The autonomic nervous system (ANS) regulates many physiological processes, since organs receive reciprocal input from the parasympathetic and sympathetic branches of ANS to maintain homeostasis. Dysregulation of the ANS can occur due to disorders or injury, including diabetes, sepsis, spinal cord injury, and other conditions. Development of quantitative biomarkers of ANS function can yield novel insights to such dysregulation and provide a useful tool to diagnose and assess the severity of these disorders. Such a metric was extracted here from physiological signals obtained by clinically standard techniques during controlled autonomic testing. Nineteen healthy, able-bodied individuals attended four sessions, each consisting of five autonomic tests; non-invasive sensors were used to capture continuous physiological data, including electrocardiography (ECG), blood pressure (BP), respiration, galvanic skin response, and pupil diameter. Raw ECG and BP signals were used to calculate heart rate, mean arterial pressure, and a heart rate variability measure, root mean square of successive differences (RMSSD) between heartbeats. Calculated signals were averaged by test and recording modality, and a template matching method was developed to characterize individual responses by determining features that represented duration and amplitude scales compared to the average response. As measured by the derived index, sympathetic amplitude responses significantly increased with higher body mass index among participants. Longer and larger sympathetic and longer parasympathetic responses were observed in afternoon testing, but larger parasympathetic responses were measured in morning testing sessions. Quantifying ANS function can provide crucial information about disease presence or intensity and treatment efficacy.

## P7. Tumor Treating Fields (TTFields): An innovative bioelectronic modality in oncology

### William F. Doyle**,** SB, MBA

#### Novocure, Portsmouth, New Hampshire, US

TTFields are frequency-specific, alternating electrical fields developed for the treatment of cancer. They are delivered non-invasively to the tumor region using a home-use medical device. TTFields’ mechanism of action is based on exertion of directional forces on polarizable microtubules, interfering with the assembly of the normal mitotic spindle. Such interference results in mitotic arrest, ultimately leading to cell death. To date, FDA has approved TTFields for recurrent and newly-diagnosed glioblastoma, and for mesothelioma.

The only frequently-reported adverse event related to TTFields is skin irritation underneath the transducer arrays applied to the skin. Since their mechanism of action suggests anti-mitotic activity in many other solid tumors, TTFields have been successfully investigated in preclinical and pilot studies in additional cancers. They are currently investigated in phase 3 trials in pancreatic, lung and ovarian cancers, and in brain metastasis. Pilot trials are ongoing in gastric and liver cancers.

The establishment of TTFields as a new treatment modality in oncology involved extensive multi-disciplinary research and development activities. The tight collaboration between physicians, biologists, physicists and engineers enabled the concept of targeting cancer cells with electric fields to become a therapy available at the clinic. This same collaboration continues to move the technology forward, with novel hardware and software advancements being implemented these days. They include more sophisticated design and materials, as well as treatment planning software for further improvements in treatment efficacy. These advancements make TTFields a more personalized modality, tuned to target the disease of an individual patient in the most effective way.

## P8. Targeted peripheral focused ultrasound stimulation attenuates obesity induced metabolic and inflammatory dysfunctions

### Tomás S. Huerta^1,2^, Alex Devarajan^1^, Tea Tsaava^1^, Arvind Rishi^3^, Victoria Cotero^5^, Christopher Puleo^5^ Jeffrey Ashe^5^, Thomas R. Coleman^1^, Eric H. Chang^1,2,4^, Sangeeta S. Chavan^1,2,4^, Kevin J. Tracey^1,2,4^

#### ^1^Institute of Bioelectronic Medicine, Feinstein Institutes for Medical Research, Northwell Health, Manhasset, New York, US; ^2^Donald and Barbara Zucker School of Medicine at Hofstra/Northwell, Hempstead, New York, US; ^3^Department of Pathology and Laboratory Medicine, Hofstra Northwell School of Medicine, Lake Success, New York, US; ^4^The Elmezzi Graduate School of Molecular Medicine, Manhasset, New York 11030, US; ^5^GE Global Research Center, Niskayuna, New York, US

##### **Correspondence:** Kevin J. Tracey

Obesity is associated with increased mortality and significant comorbidities, including insulin resistance, metabolic syndrome, and cardiovascular disease. Chronic low-grade inflammation is a hallmark of the pathophysiology of obesity. Recent studies have utilized noninvasive peripheral focused ultrasound stimulation (pFUS) to the liver to improve glucose metabolism in endotoxemia. Here we reasoned that pFUS of the liver may alleviate diet-induced obesity related inflammation and other comorbidities. After 16 weeks, obese mice that received daily hepatic pFUS, from weeks 9 to 16, had significantly decreased body weights compared to sham-stimulated mice on the same diet (*P* < 0.01, 2-way RM ANOVA). pFUS also significantly reduced adipokine levels (*P* = 0.05, 2-way ANOVA) and circulating lipids (*P* < 0.05, 2-way ANOVA) in obese mice. Hepatic pFUS significantly reduced liver inflammation observed with reduced cytokine levels (*P* < 0.001, 1-way ANOVA) and leukocyte infiltration (*P* < 0.0001, Kruskal-Wallis H test). Our findings demonstrate the efficacy of end-organ targeted focused ultrasound to the porta hepatis for alleviating diet-induced obesity and improving several aspects of metabolic health in mice. Our results highlight the possibility that hepatic focused ultrasound might be used as a novel noninvasive treatment for obesity.

## P9. Spatial multiplexing of fluorescent reporters for dynamic imaging of signal transduction networks

### Shannon L. Johnson*, MSc^1^, Changyang Linghu*, PhD^1^, Pablo A. Valdes, MD, PhD^1,2^, Or A. Shemesh, PhD^1^, Won Min Park, PhD^3^, Demian Park, PhD^1^, Kiryl D. Piatkevich, PhD^1^, Asmamaw T. Wassie, PhD^1^, Yixi Liu, BS^1^, Bobae An, PhD^1^, Stephanie A. Barnes, PhD^1^, Orhan T. Celiker, MSc^1^, Chun-Chen Yao, BSc^1^, Chih-Chieh (Jay) Yu, BSE^1^, Ru Wang, PhD^1^, Katarzyna P. Adamala, PhD^4^, Mark F. Bear, PhD^1^, Amy E. Keating, PhD^1^, and Edward S. Boyden, PhD^1^

#### ^1^Massachusetts Institute of Technology, Cambridge, Massachusetts, US; ^2^Harvard Medical School, Brigham and Women's Hospital, Boston, Massachusetts, US; ^3^Kansas State University, Manhattan, Kansas, US; ^4^University of Minnesota, Minneapolis, Minnesota, US

##### **Correspondence:** Edward S. Boyden

* These authors contributed equally

Bioelectronic medicine depends on a detailed understanding of how signals within and between neurons, glia, and immune cells interact, along with their impact on organ health. A toolset that enables the simultaneous imaging of many signals in real-time would allow such signaling maps to be obtained, but classical imaging is limited by the number of fluorescent colors that do not overlap. We have invented a way to map potentially arbitrary numbers of signals within a single living cell by fusing a fluorescent reporter to a pair of self-assembling peptides. The reporters stably clustered within cells at random points, distant enough to be resolved by a microscope but close enough to spatially sample the relevant biology. The clusters, which we call signaling reporter islands (SiRIs), can be modularly designed to permit existing fluorescent reporters to be efficiently adapted for simultaneous measurement of many nodes in a signal transduction network within single living cells. We created SiRIs for indicators of second messengers and kinases and used them to image up to 5 signals at once in a single living neuron. Applied to hippocampal neurons in both culture and brain slice, we discovered relationships between the speed of calcium signaling and the amplitude of PKA cellular signaling, upon receiving a cAMP-driving cellular input. Thus, SiRIs will enable understanding the relationships between the components of molecular decision-making in immune cells, neurons, and other cell types. Being fully genetically encoded, SiRIs will hopefully become an easy-to-use component of the bioelectronic medicine development toolbox.

## P10. Development and Validation of a 7-Day Survival Calculator for Hospitalized Patients with COVID-19

### Todd J. Levy^1^, MS; Safiya Richardson^23^, MD, MPH; Kevin Coppa^4^, BS; Douglas P. Barnaby^23^, MD, MSc; Thomas McGinn^23^, MD, MPH; Lance B. Becker^12^, MD; Karina W. Davidson^23^, PhD, MASc; Stuart L. Cohen^23^, MD; Jamie S. Hirsch**^234^, MD, MA, MSB; Theodoros P. Zanos**^12^, PhD, And the Northwell & Maimonides COVID-19 Research Consortium; Hannah Bodenstein^5^, MBA, BSN, RN; Shubham Debnath^1^, PhD; Andrew J. Dominello^3^, BA; Louise Falzon^3^, BA, PGDipInf; Michael Gitman^2^, MD; Jay M. Goldstein^6^, MBA; Crystal Herron^3^, PhD; Eun-Ji Kim^23^, MD, MS; Lawrence Lau^2^; Zachary S. Lockerman^5^, MD, MBA, FACG, CPE; Alexander Makhnevich^2^, MD; Jazmin N. Mogavero^3^, MA; Ernesto P. Molmenti^23^, MD, PhD, MBA, FACS; Marc d. Paradis^7^, SM; Viktor Tóth^1^, MSc; Avantika Vardhan^1^, PhD;

#### ^1^Institute of Bioelectronic Medicine, Feinstein Institutes for Medical Research, Northwell Health, Manhasset, NY US; ^2^Donald and Barbara Zucker School of Medicine at Hofstra/Northwell, Northwell Health, Hempstead, NY US; ^3^Institute of Health Innovations and Outcomes Research, Feinstein Institutes for Medical Research, Northwell Health, Manhasset, NY US; ^4^Department of Information Services, Northwell Health, New Hyde Park, NY USA; ^5^Maimonides Medical Center, Brooklyn, NY US; ^6^Management Information Systems, Maimonides Medical Center, Brooklyn, NY USA; ^7^Department of Data Strategy & Ventures, Northwell Health, Manhasset, NY US

##### **Correspondence:** Jamie S. Hirsch; Theodoros P. Zanos

**Co-senior authors with equal contribution

Chinese studies reported predictors of severe disease and mortality associated with coronavirus disease 2019 (COVID-19). A generalizable and simple survival calculator based on objective data from US patients hospitalized with COVID-19 has not yet been introduced. Our goal was to develop and validate a clinical tool to predict 7-day survival in patients hospitalized with COVID-19. We used electronic health record data of 13027 adult patients hospitalized with a confirmed diagnosis of COVID-19 from thirteen acute care hospitals in the New York City area, 12 of which were part of Northwell Health. Demographic, laboratory, clinical, and outcome data were extracted from the electronic health records. Optimal predictors and performance were identified using least absolute shrinkage and selection operator (LASSO) regression with receiver operating characteristic curves and measurements of area under the curve (AUC). Serum blood urea nitrogen, age, oxygen saturation, serum sodium, red cell distribution width, and absolute neutrophil count were identified as the 6 optimal of 41 possible predictors of survival. These factors constitute the NOCOS (Northwell COVID-19 Survival) Calculator. Performance in the prospective validation and external validation were marked by AUCs of 0.80 ([0.75, 0.84] 95% CI), and 0.78 ([0.75, 0.80] 95% CI), respectively. Collectively, the NOCOS Calculator uses 6 factors routinely available at hospital admission to predict 7-day survival for patients hospitalized with COVID-19, and outperforms existing calculators like SOFA (p < 0.05 internal prospective validation) that are not specifically designed for COVID-19. The calculator is publicly available at https://feinstein.northwell.edu/NOCOS.

## P11. Trigeminal nerve stimulation induced hemodynamic oscillation confers neuroprotection for combined traumatic brain injury and hemorrhagic shock

### Kevin A. Shah, MD^1,2^, Anup N. Sonti, BS^1,2^, Keren Powell, BA^1^, Yi-Chen Wu, MS^1^, Wayne Chaung, PhD^3^, Ping Wang, MD^3^, Weng-Lang Yang, PhD^4^, Lance B. Becker, MD^5^, Raj K. Narayan, MD^1,2^, Chunyan Li, PhD^1,2^

#### ^1^Translational Brain Research Laboratory, Institute of Bioelectronic Medicine, The Feinstein Institutes for Medical Research, Manhasset, NY, US; ^2^Department of Neurosurgery, Zucker School of Medicine at Hofstra/Northwell, Hempstead, NY, US; ^3^Center for Immunology and Inflammation, The Feinstein Institutes for Medical Research, Manhasset, NY, US; ^4^Department of Radiation Oncology, Albert Einstein College of Medicine, Bronx, NY, US; ^5^Department of Emergency Medicine, Donald and Barbara Zucker School of Medicine at Hofstra/Northwell, Manhasset, NY, US

##### **Correspondence:** Chunyan Li

**Background**

Combined traumatic brain injury (TBI) and hemorrhagic shock (HS) (TBI+HS), is associated with high levels of morbidity and mortality. The current resuscitative approach to TBI+HS involves intravascular volume expansion, vasopressors, and oxygen supplementation which requires access to medical care and yet inadequately address the severe hypoperfusion that distinguishes this condition. For decades, large spontaneous oscillations in blood pressure (BP) and cerebral blood flow (CBF) have been observed in individuals with high tolerance to hypovolemia. These hemodynamic waves represent an endogenous physiologic mechanism that improves cerebral perfusion and confers protection to cerebral tissue. In the present study, we explored the ability of electrical trigeminal nerve stimulation (TNS) to mimic the hemodynamic oscillations shown in individuals with high hypovolemia tolerance, and investigated its beneficial effects in a rat model of TBI+HS.

**Methods and Results**

TBI+HS was induced by controlled cortical impact, and subsequent pressure-controlled HS (27±2 mmHg) for 35 minutes in male Sprague-Dawley rats. TNS induced large, synchronized oscillations in BP and CBF. These bio-mimicked hemodynamic oscillations attenuated the severity of brain injury, improved the survival rates, and facilitated functional recovery after TBI+HS. Secondary oscillations of P_br_O_2_ reduced hypoxic stress as confirmed by HIF-1a expression in the hippocampus. Furthermore, non-specific TNS did not induce hemodynamic oscillations and did not show any the beneficial effects in this animal model of TBI+HS.

**Conclusions**

Bio-mimicked synchronized fluctuations of BP and CBF, and secondary oscillations of P_br_O_2_ can be induced by specifically calibrated TNS to provide rapid pre-hospital treatment and improve survival outcomes following TBI+HS.

## P12. *In vivo* detection of VNS-elicited norepinephrine release in the spleen in real-time

### Ibrahim Mughrabi MD, PhD^*^, Naveen Jayaprakash**,** PhD^*^, Adam Abbas, BSc, Yousef Al-Abed, PhD, Stavros Zanos MD, PhD

#### Institute of Bioelectronic Medicine, The Feinstein Institutes for Medical Research, Manhasset, New York, US

##### **Correspondence:** Stavros Zanos

^*^These authors contributed equally to this work

Vagus nerve stimulation (VNS) is an emerging bioelectronic therapy with the potential to treat a wide range of conditions in which inflammation is implicated. Anti-inflammatory actions of VNS require norepinephrine (NE) release into the spleen by post-ganglionic neurons in abdominal ganglia. Real-time detection of NE could provide a marker to calibrate and optimize VNS anti-inflammatory effects in individual subjects. Here, we describe a method to study in vivo VNS-elicited NE release in the spleen, using fast scan cyclic voltammetry (FSCV). Initially, NE oxidation potential was determined in vitro to be approximately 0.6 V, using standard cycling parameters. In experiments in anesthetized mice instrumented with ECG, a carbon fiber voltammetry electrode was inserted into the exposed spleen. VNS was delivered using a bipolar cuff electrode placed on the left cervical VN. Short trains of VNS (2-10 s duration, 100 μs pulse width) induced a peak oxidation current at 0.6 V indicating NE release at pulsing frequencies >5Hz (N=8 animals). NE currents were detected <10 s after VNS onset, peaked at 20-30 s, and returned to baseline within 1 to 10 min (N=5). Peak NE oxidation current was proportional to VNS intensity and to the concomitant VNS-elicited reduction in heart rate (N=3). These preliminary findings indicate that FSCV may be a reliable method for the real-time measurement of NE release in the spleen, thereby providing an in vivo marker of anti-inflammatory effects of VNS.

## P13. The effect of focused ultrasound neuromodulation of the liver on glucose metabolism in healthy mini-pigs

### Qanud K^1^, Tomaio J^1^, Song W^1^, Hagve M^2^, Gjessing P^2^, Al-Abed Y^1^, Puleo C^3^, Zanos S^1^

#### ^1^Institute of Bioelectronic Medicine, Feinstein Institutes for Medical Research, Manhasset, NY, US; ^2^UiT The Arctic University of Norway, Dept. of Clinical Medicine, Tromso, NO; ^3^GE Global Research, Niskayuna, New York, US

##### **Correspondence:** Zanos S

Recently, focused ultrasound (FUS) neuromodulation was shown to have organ-specific effects in rodents undergoing acute inflammation, including a decrease in blood glucose levels when targeting the liver (Cotero V *et al*, Nature Comm 10: 952, 2019). It is unclear whether FUS has similar metabolic effects in large animals with similar anatomy and physiology to humans, in the absence of inflammation. In healthy mini-pigs, we validated whether FUS targeting the porta hepatis (PH) of the liver could lead to changes in glucose metabolism. First, in a series of hyperinsulinemic-euglycemic clamp experiments, we used different insulin infusion rates (IIRs) and determined the glucose infusion rate that achieved an euglycemic equilibrium (eGIR), a measure of insulin sensitivity independent of blood glucose levels. We found that measures of insulin sensitivity were larger at low IIRs (“unsaturated”) than at high IIRs (“saturated”). Then, in a second series of experiments, eGIR was determined during low and high IIRs, before and after 4-min-long FUS of the PH. FUS during saturated IIR euglycemia conditions (7 experiments, in 2 animals) resulted in increase of eGIR in 1 experiment, decrease in 1 experiment and no changes in 5 experiments. FUS delivery during unsaturated IIR euglycemia (5 experiments in 2 animals) resulted in increase of eGIR in 4 experiments and no change in 1 experiment. No significant changes in liver tests and other biochemical and hematological parameters were associated with FUS. In conclusion, these preliminary results suggest that, in a swine model, FUS neuromodulation of the PH may increase insulin sensitivity without inducing liver injury.

## P.14 TRPA1 is necessary for IL-1β induced vagus nerve activity

### Harold A. Silverman**,** PhD^1^, Eric Chang, PhD^1^, Manojkumar Gunasekaran, PhD^1^, Tea Tsaava, MD^1^, Meghan Addorisio, BA,^1^, Tomas S. Huerta, BS^1,2^, Andrew Stiegler, BS^1,2^, Adam M. Kressel, MD, PhD^1,3,4^, Jian Hua Li, MS^1^, Qing Chang, PhD^1^, Qiong Zeng, PhD^1^, Adrian Chen^1^, Valentin A. Pavlov, PhD^1,2,3,5^, Ulf Andersson, MD, PhD^5^, Kevin J. Tracey, MD^1,2,4,5,^, Sangeeta S. Chavan, PhD^1,2,4,5^

#### ^1^Laboratory for Biomedical Sciences, Institute for Bioelectronic Medicine, Feinstein Institutes for Medical Research, Northwell Health, Manhasset, New York, US; ^2^Donald and Barbra Zucker School of Medicine at Hofstra/Northwell, Manhasset, New York, US; ^3^Department of Surgery, Northshore University Hospital, Northwell Health, Manhasset, New York, US; ^4^The Elmezzi Graduate School of Molecular Medicine, Northwell Health, Manhasset, New York, US; ^5^Department of Women’s and Children’s Health, Karolinska Institute, Karolinska University Hospital, Stockholm, SE

##### **Correspondence:** Kevin J. Tracey; Sangeeta S. Chavan

Sensory vagus nerve fibers transmit information in the form of action potentials to the central nervous system to maintain immunological homeostasis. Pro-inflammatory molecules including LPS, TNF, and IL-1β induce cytokine specific electrophysiological changes in vagus nerve signaling. In addition, these pro-inflammatory molecules directly activate nodose ganglion neurons, the location of sensory vagus nerve cell bodies. Previous studies have shown cytokines have a direct impact on cation channels TRPV1 and TRPA1, which are expressed on sensory fibers of the vagus nerve. Reasoning that these cation channels may play a role in vagus nerve mediated cytokine signaling we explored TRPA1’s role in IL-1β induced vagus nerve activity. Extra neural electrical activity was recorded from the cervical vagus nerve in response to i.p. administration of IL-1β. IL-1β induces a significant increase in vagus nerve activity in wild type mice, however this response was ablated in whole body TRPA1 KO mice as well as neuronal specific TRPA1 KO mice (SynCre-TRPA1flox). Using calcium imaging and whole cell patch clamp recordings, we observed nodose ganglion neurons deficient in TRPA1 do not respond to IL-1β application. In addition, IL-1β induced calcium influx was inhibited by administration of the TRPA1 antagonist AM0902. These results elucidate a novel role for the TRPA1 cation channel as a necessary component for IL-1β induced vagus nerve signaling.

## P15. A micro-fabricated polyimide electrode with Ir/IrO_x_ electrode sites for chronic mouse vagus nerve interface

### Tao Sun**,** PhD^1^, Joanne Peragine, BS^1^, Christine Crosfield, BS^1^, Romil Modi, MS^1^, Rohit Sharma, MS^2^, Maria Fernanda Lopez, BS^1^, Loren Rieth, PhD^1^

#### ^1^Institute for Bioelectronic Medicine, Feinstein Institutes for Medical Research, Manhasset, NY, US; ^2^Electrical and Computer Engineering, University of Utah, Salt Lake City, Utah, US

##### **Correspondence:** Loren Rieth

Micro-fabricated polyimide (PI)-based neural electrodes have been actively pursued with innovations including electrode materials, architectures, microfabrication processes, and seamless integration with nervous tissues. Mouse vagus nerve (~100 μm) is a key therapeutic and research target in the field of bioelectronic medicine. However, the technology to interface delicate nerves for chronic endpoints has been a challenge. In this study, a PI flexible neural *Flex* electrodes with Ir/IrO_x_ electrode sites was fabricated via a microelectromechanical system (MEMS) process, and an integration strategy was developed for the chronic interface with the mouse vagus nerve, including an epoxy reinforcement for the joints between electrode bonding pads and platinum wire leads, and a thermoforming process to shape the *Flex* electrodes for intimiate contact with the nerve. The electrochemical properties of the flex electrode, such as impedance and charge storage capacity (CSC), were characterized using electrochemical impedance spectroscopy (EIS) and cyclic voltammetry (CV), respectively. The impedance of the *Flex* electrode at 1 kHz was as low as 617.15 ± 13.17 Ω. The impedance spectra were fitted with an equivalent circuit model to clarify the electrode-electrolyte interface. Compared to the state-of-the-art with respect to the neural electrode with IrO_x_ stimulation sites, the *Flex* electrode developed in this study has the CSC of 17.02 ± 3.04 mC/cm^2^. This study represents one of dedicated efforts to develop the chronic mouse vagus nerve interface, using microfabricated electrodes.

## P16. Single-axon level segmentation and feature extraction pipeline combining traditional and deep learning computer vision algorithms

### Viktor Tóth, Naveen Jayaprakash, Adam Abbas, Moontahinaz Rob, Ariba Khan, Stavros Zanos, Theodoros P. Zanos

#### Institute of Bioelectronic Medicine, Feinstein Institutes for Medical Research, Northwell Health, Manhasset, NY, US

##### **Correspondence:** Stavros Zanos; Theodoros P. Zanos

Quantitative descriptions of the morphology and structure of peripheral nerves is important for bioelectronic and brain-machine interface devices. While histological procedures and microscopy techniques yield high-resolution detailed images of individual axons, automated methods to extract relevant information at the single-axon level are not widely available. We implemented a computer vision segmentation algorithm that allows for subsequent feature extraction in immunohistochemistry (IHC) images of peripheral nerves at the single fiber scale. These features include short and long cross-sectional diameter, area, and perimeter, thickness of surrounding myelin and polar coordinates of single axons within a nerve or nerve fascicle. To improve the performance of our computer vision algorithm, we generated a dataset of segmented immunohistochemical images and trained a deep convolutional neural network (Mask R-CNN) on instance segmentation. The dataset of segmented examples was produced by the original algorithm and subsequently refined by human annotators, yielding a significant 4-fold decrease in human labor compared to the process of manual annotation from scratch. A combined solution of the original algorithm and the deep convolutional neural network achieved a single-axon detection accuracy of 98%, as opposed to 82% using the traditional computer vision algorithm alone, on a held-out nerve section of 36 fascicles of the swine cervical vagus.

## P17. Optogenetic activation of specific vagal fiber populations to modulate serum cytokines

### Téa Tsaava^1^, Sangeeta S. Chavan^1,2^, Kevin J. Tracey^1,2^, Eric H. Chang^1,2^

#### ^1^Institute of Bioelectronic Medicine, Feinstein Institutes for Medical Research, Northwell Health, Manhasset, New York, US; ^2^Donald and Barbara Zucker School of Medicine at Hofstra/Northwell, Hempstead, New York, US

##### **Correspondence:** Eric H. Chang

Electrical stimulation of the vagus nerve inhibits expression of proinflammatory cytokines, such as tumor necrosis factor (TNF), through a neural circuit termed the inflammatory reflex. To determine whether vagus fiber populations could be selectively activated to control inflammatory and physiological function, we created ChAT-ChR2-eYFP, TRPV1-ChR2-eYFP, and VGlut2-ChR2-eYFP mice expressing light-sensitive channelrhodopsin-2 (ChR2) in specific neuronal and fiber populations. Utilizing in situ cervical vagus nerve preparations, we optogenetically stimulated vagal fibers (opto-VNS) with the following parameters: 470 nm, 1000 mA, 10 Hz, 50 ms, 5 min. After stimulation mice were then injected i.p. with lipopolysaccharide (0.3 mg/kg) to induce acute endotoxemia, then killed 90 mins later. Serum TNF was measured by ELISA. We observed that in a subset of ChAT-ChR2 mice, opto-VNS significantly decreased levels of TNF compared to sham-matched controls (t-test, *P*<0.02). Opto-VNS did not change TNF levels in TRPV1-ChR2 or VGlut2-ChR2 mice compared to sham-stimulated controls. We also found that opto-VNS induced varying degrees of transient bradycardia in all three strains: ChAT-ChR2 (heart rate pre: 495.44±21.45, during: 484.39±15.02), TRPV1-ChR2 (pre: 438.87±19, during: 357.26±29.78), VGlut2-ChR2 (Pre: 421.67±16.92, during: 353.33±27.47). Respiration rate increased during opto-stimulation in TRPV1-ChR2 (pre: 85.06±5.79, during: 157.66±24.84), and VGlut2-ChR2 (pre: 76.39±12.59, during: 108.23±15.49), but not in ChAT-ChR2 mice (pre: 93.43±16.46, during: 99.47±18.04). Our results indicate selective optogenetic activation of vagal fibers produces distinct physiological changes based on the population activated, and that opto-VNS can be used to change serum cytokine levels in acute endotoxemia.

## P18. TRPV1+ sensory nerves modulate antigen specific immune responses

### Aisling Tynan PhD^1^, Téa Tsaava, MD^1^, Manojkumar Gunasekaran PhD^1^, Kevin J. Tracey, MD^1,2,^ and Sangeeta S. Chavan, PhD^1,2^

#### ^1^Institute of Bioelectronic Medicine, Feinstein Institutes for Medical Research, Northwell Health, Manhasset, New York, US; ^2^Elmezzi Graduate School of Molecular Medicine, Manhasset, New York, US

##### **Correspondence:** Kevin J. Tracey; Sangeeta S. Chavan

The nervous system and the immune system are the two major organs of memory and host defense. Sensory neurons have been implicated in enhancing neurological memory, but whether neurons participate during immunity to novel antigens is unknown. Here, we show that TRPV1 expressing neurons are required to develop an antigen specific immune response and that direct activation of TRPV1+ signals by selective optogenetic stimulation enhances this response to novel antigen. We generated TRPV1-Cre/Lox-stop-lox diphtheria toxin A (TRPV1-DTA) mice to specifically ablate TRPV1 expressing neurons. TRPV1-DTA mice were subjected to immunization with keyhole limpet hemocyanin (KLH) and antigen-specific antibody responses were measured over 28 days. KLH-specific antibody responses were significantly blunted in TRPV1-DTA mice as compared to wild-type controls for 28-days post immunization. Next, we optogenetically activated TRPV1 neurons during immunization. TRPV1-Cre/Rosa-ChannelRhodopsin2 (TRPV1-ChR2) mice received optical stimulation on the dorsum of the paw with blue light immediately prior to a local immunization of KLH. Direct optogenetic stimulation of TRPV1+ neurons significantly increased KLH-specific antibody responses as compared to wild-type mice. Collectively these findings reveal that TRPV1-expressing sensory neurons modulate antigen-specific immune responses. This is the first genetic and selective functional evidence that nociceptors are required during immunization to produce antigen-specific antibodies.

## P19. Comparative analysis of NLP algorithms for the extraction of signs and symptoms from EHR (Electronic Health Records)

### Avantika Vardhan^1^, Kevin Coppa^2^, Paul Chung^2^, Marcia Epstein^2^, Prashant Malhotra^2^, Negin Hajizadeh^2^, Jamie Hirsch^2^, Theodoros Zanos^1^

#### ^1^Institute for Bioelectronic Medicine, Feinstein Institutes for Medical Research, Manhasset, New York, US; ^2^Northwell Health, Manhasset, New York, US

##### **Correspondence:** Theodoros Zanos

The goal of this work is to (1) create pipelines for entity data extraction using multiple medical NLP tools, (2) extract the signs and symptoms from an EHR (Electronic Health Records) corpus via this pipeline, and (3) compare results from the pipeline with manual annotations by clinicians. The NLP tools chosen for comparison in this particular study are CTAKES and CLAMP. Software pipelines based on Java and Python were built to process health records via these tools. Signs and symptoms that were extracted via these tools as XMI files were then standardized using Python-based data extraction techniques. The resulting signs and symptoms were further classified as positive (present), and negative (absent) by the tools. Clinicians also created a manually annotated ground truth to enable quantitative analysis of accuracy of each tool. The Jaccard (dice coefficient) was used to determine the degree of overlap between the 2 NLP tools and 3 annotators, resulting in a 2-by-3 matrix of values. For each tool-annotator comparison, the Jaccard coefficient was computed on a per-record basis and then averaged. For a single record, the overlap across each sign/symptom entity was averaged to get a record-level score. As a result, the NLP tools were evaluated against a manual ground truth and baseline performance metrics were extracted. In the future, this work could enable analysis and accuracy comparison of various NLP tools for the purpose of a variety of entity recognition purposes from health records, as was done with signs and symptoms in the case above.

## P20. Development of fully-implantable wireless stimulators for small animal bioelectronic medicine research

### Jason Wright, MEng, Jason Wong, MSc, Ibrahim Mughrabi, MD, PhD, Naveen Jayaprakash, PhD, Stavros Zanos, MD, PhD, and Timir Datta-Chaudhuri, PhD

#### Institute of Bioelectronic Medicine, Feinstein Institutes for Medical Research, Manhasset, New York, US

##### **Correspondence:** Stavros Zanos; Timir Datta-Chaudhuri

The development of modern bioelectronic therapeutics frequently relies upon *in vivo* studies of the physiological effects of neurostimulation. Numerous disease models available in small rodents provide ideal research settings; however, developing implants for small animals with cm-sized anatomies poses a technological challenge. Size constraints derive from limits on the energy capacity of ultra-miniature batteries, integration of electrical circuits, and the need for resilient biocompatible packaging. While commercial medical devices offer multi-year lifetimes, devices for animal research typically target shorter lifetimes of weeks to months in order to reduce their volume, mass, and cost.

Previously, our lab has demonstrated a novel constant-current neurostimulator providing basic control of stimulation parameters. We present an updated, fully-wireless design that utilizes near-field communication for dynamic control, enabling precise control of stimulation and more sophisticated experiments, as well as improved output characteristics. The device offers 0-3 mA amplitude, as short as 100 μs pulses, and frequency from DC to 10 kHz. Data from acute and chronic experiments validate the core functionality in the mouse model. Preliminary results suggest a usable lifetime longer than one month.

## P21. HMGB1 mediates neurogenic inflammation

### Huan Yang**,** PhD^1^, Qiong Zeng, PhD^1^, Harold Silverman, PhD^1^, Sam George, BA^1^, Meghan E. Addorisio, BS^1^, Jianhua Li, MS^1^, Téa Tsaava, MD^1^, Ulf Andersson, MD, PhD^2^, Valentin A. Pavlov, PhD,^1,3,4^, Eric H. Chang, PhD^1,3,4^, Sangeeta S. Chavan, PhD ^1,3,4^, and Kevin J. Tracey, MD^1,3,4^

#### ^1^Institute for Bioelectronic Medicine, Feinstein Institutes for Medical Research, Northwell Health, Manhasset, New York, US; ^2^Department of Women’s and Children’s Health, Karolinska Institute, Karolinska University Hospital, Stockholm, SE; ^3^Elmezzi Graduate School of Molecular Medicine, Feinstein Institutes for Medical Research, Northwell Health, Manhasset, New York, US; ^4^Donald and Barbara Zucker School of Medicine at Hofstra University, Northwell Health, Hempstead, New York, US

##### **Correspondence:** Sangeeta S. Chavan; Kevin J. Tracey

Vertebrates control homeostasis during infection or injury by balancing the activity of pro- and anti-inflammatory responses. Reflex neural circuits inhibiting inflammation are well established, but the role of neurons stimulating inflammation is not clear. High mobility group box 1 (HMGB1), an established mediator in both infectious disease and sterile injury, is abundantly expressed in neurons. To elucidate the role of neuron-derived HMGB1 in neurogenic inflammation and nociceptive response, we generated neuronal-specific conditional HMGB1 knockout mice. Selective silencing of HMGB1 in neuronal tissues attenuates neurogenic inflammation in animal model of sciatic nerve chronic constriction injury (CCI). Genetic silencing of neuronal HMGB1 attenuated local HMGB1 expression, as well as CXCL1, IL-18 and TNF levels in the inflamed paws as compared to HMGB1 flox or wild type control mice. Sciatic nerve CCI-induced hyperalgesia (assessed by mechanical hypersensitivity via the von Frey test) was inhibited in the neuronal-HMGB1 deficient mice. Administration of anti-HMGB1 monoclonal antibodies conferred significant protection against sciatic nerve CCI-induced mechanical and thermal hyperalgesia. Furthermore, in collagen antibody-induced arthritis (CAIA), genetic silencing of neuronal HMGB1 significantly inhibited CAIA-induced joint edema formation, hyperalgesia and inflammatory cell infiltration as compared to CAIA in HMGB1 flox and wild type controls. Collectively, these findings reveal a previously unrecognized critical role for neuronal HMGB1 in initiating inflammation and nociceptive responses in the sciatic nerve CCI and CAIA models.

## S1. Optoelectronic medicine – nongenetic means of nerve cell regulation with light

### Eric Daniel Głowacki, PhD^1,2^

#### ^1^Laboratory of Organic Electronics, ITN Campus Norrköping, Linköping University, SE-60174, Norrköping, SE; ^2^Central European Institute of Technology, Brno University of Technology, Purkynova 123, 61200 Brno, CZ

A great demand exists for minimally-invasive neuromodulation technologies to enable next-generation bioelectronic medicine. We report on our developments of ultrathin (opto)electronic devices for neurostimulation. All of these devices rely on far red/near infrared irradiation in the tissue transparency window to actuate nanoscale organic semiconductor components. Our flagship technology is the organic electrolytic photocapacitor (OEPC) – a device that mimics biphasic current-pulse neurostimulation and thus transduces an optical signal into directly-evoked action potentials in neurons. These devices are not only wireless, but also 100-1000 times thinner than existing technologies. We will discuss chronic implants capable of stimulating peripheral nerves (sciatic and vagus) when actuated from outside of the body using diode lasers. Light power can be safely and effectively transmitted to implants up to 15 mm below the skin surface. We have observed stable operation in rodent models for at least 100 days. We believe that the combination of deep red light and ultrathin photovoltaic devices can account for a new paradigm in wireless bioelectronic medicine.

## S2. Ultracompliant hydrogel electronics for electrophysiological monitoring

### Christopher J Bettinger, PhD

#### Carnegie Mellon University, Pittsburgh, PA, US

Reliable recording and modulation of excitable tissue using implantable electronic devices have implications in diagnosing and treating many types of diseases. The advent of flexible electronics has enabled new concepts in interfacing devices with soft tissues. However, to date, most flexible electronics achieve mechanical compliance by using substrates composed of thin curable resins or elastomers. These materials are suboptimal for tissue interfacing because they: (1) exhibit Young’s moduli that are orders of magnitude larger than many excitable tissues, such as peripheral nerves; (2) are difficult to integrate with hydrated tissue in vivo. Here I will present recent advances in materials and fabrication from our lab to address current limitations in flexible electronics. Specifically, the synthesis and formulation of adhesive hydrogels and transfer printing to create ultracompliant peripheral nerve interfaces will be described. Details regarding the in vitro and in vivo performance of ultracompliant electronics will be presented. Future prospective applications for this concept will also be highlighted, too.

## S3. Communicating with the nervous system for the cure of diseases

### Martin Schuettler

#### CorTec GmbH, Freiburg, DE

CorTec develops a chronically implantable brain-computer interface called “Brain Interchange” for bi-directional communication with the brain. The system consists of various components: cortical grid electrodes, implantable electronics protected by ceramic hermetic package and body-external transceiver that communicates wirelessly with the implant and also wirelessly supplies the implant with energy via an inductive link. The system streams neural data from 32 electrode contacts to a computer where the data is processed and stored. Based on the incoming neural data, algorithms on the computer can take decisions on therapeutic actions, e.g. sending a stimulation command wirelessly to the implant which then delivery electrical pulses via the implanted electrodes to the brain, modulating brain activity. This closed-loop system can be used for patient-individual and situation-specific brain therapy, treating a variety of central nervous system related disorders. The system can also be used as bi-directional communication tool between brain and computer.

Besides the development of Brain Interchange as a platform for central nervous system related therapy discovery and brain-machine interface studies, CorTec provides the innovative technology that permitted building the individual components of Brain Interchange to others. Especially the technology for producing laser-micromachined neural electrodes that permit high production precision while using only traditional implant materials such as medical grade silicone rubber and PtIr noble metal foil is applied to wide range of applications, also in the field of spinal cord stimulation and peripheral nerve interfacing, in preclinical as well as in clinical studies.

Another technology made available to others is a ceramic hermetic package for electronic implant circuits that permits a very high number of electrical feedthroughs (100s) while providing excellent hermeticity.

With these and other technologies, CorTec is dedicated to improving the field of neurotechnology and neural therapy discovery.

## S4. Targeting populations of fibers in clinically-relevant vagus nerve stimulation

### Stavros Zanos, MD, PhD

#### Institute of Bioelectronic Medicine, Feinstein Institutes for Medical Research, Manhasset, NY, US

Current clinical vagus nerve stimulation (VNS) therapies rely on engaging vagal fibers in a relatively uncontrolled manner, sometimes resulting in sub-therapeutic doses to targeted organs and side-effects from non-targeted organs. We discuss our efforts to quantify engagement of vagal fibers and end-organs, and strategies to target sub-populations of fibers in an individual subject basis. Our work is carried out in different animal models of VNS with clinically-preferred, non-penetrating cuff electrodes. Such methods for “targeted vagal neuromodulation” may contribute to using VNS to treat diseases in which hemodynamic state can easily be compromised, like heart failure and pulmonary hypertension.

## S5. Neuroimmune Cardiovascular Interfaces form Atherosclerosis Brain Circuits

### Sarajo Mohanta^1^, Li Peng^2^, Yuanfang Li^1^, Changjun Yin^1^, Shu Lu^1^, Ting Sun^1^, Lorenzo Carnevale^3^, Marialuisa Perrotta^4^, Benjamin Förstera^5^, Karen Stanic^5^, Borhan Saeed^6^, Zhe Ma^1^, Raimondo Carnevale^3^, Desheng Hu^7^, Mariaelvy Bianchini^1^, Piotr Szczepaniak^8^, Richard Nosalski^8^, Fabio Pallante^3^, Michael Beer^9^, Donato Santovito^1^, Ali Ertürk^5^, Thomas Mettenleiter^10^, Barbara Klupp^10^, Remco Megens^1^, Sabine Steffens^1^, Jaroslav Pelisek^11^, Hans-Henning Eckstein^11^, Livia Habenicht^12^, Giuseppe D’Agostino^13^, Tomasz Guzik^8^, Peder Olofsson^14^, Christian Weber^1^, Giuseppe Lembo^3,4^, Daniela Carnevale^3,4^, and Andreas Habenicht^1^

#### ^1^Institute for Cardiovascular Prevention, Ludwig-Maximilians-University, Munich, Germany; ^2^Department of Cardiovascular Internal Medicine, Guizhou University of Traditional Chinese Medicine, Guizhou, China; ^3^Department of Angiocardioneurology and Translational Medicine, IRCCS Neuromed, Pozzilli, Italy; ^4^Department of Molecular Medicine, Sapienza University of Rome, Rome, Italy; ^5^Institute for Stroke and Dementia Research, Ludwig-Maximilians-University, Munich, Germany; ^6^Department of Medicine V, University of Heidelberg, Heidelberg, Germany; ^7^Helmholtz Zentrum München, Munich, Germany; ^8^Institute of Cardiovascular and Medical Sciences, University of Glasgow, Glasgow, United Kingdom; ^9^Department for Information Technology, Jena University Hospital, Jena, Germany; ^10^Institute of Molecular Virology and Cell Biology, Friedrich-Loeffler-Institut, Greifswald, Germany; ^11^Department for Vascular and Endovascular Surgery, Technical University of Munich, Munich, Germany; ^12^II. Medizinische Klinik und Poliklinik; Technical University of Munich, Munich, Germany; ^13^School of Medical Sciences, University of Manchester, Manchester, United Kingdom; ^14^Center for Bioelectronic Medicine, Karolinska University Hospital, Stockholm, Sweden

##### **Correspondence:** Sarajo Mohanta

Atherosclerosis is a chronic nonresolving inflammatory disease of medium- and large-sized arteries causing heart attacks and strokes. As atherosclerotic plaques in the inner intimal layer and aortic media of these arteries lack nerve fibers, the impact of innervation on atherosclerosis remains unknown. However, nerves use the adventitia as their primary conduit to reach target tissues. We and others characterized artery tertiary lymphoid organs (ATLOs) in the aorta adventitia of apolipoprotein E knock-out mice and also found such aggregates in human diseased coronary arteries and other arterial tree segments. In view of these observations, we postulated that the peripheral nervous system may interact with diseased arteries via adventitial immune cells. We found in aged hyperlipidemic mice and in human atherosclerotic tissues that widespread neuro-immune-cardiovascular-interfaces (NICIs) establish atherosclerosis-brain circuits (ABCs) capable of sensing and affecting atherosclerosis: (i) ATLOs interact with the peripheral nervous system (PNS) by stimulating axon growth; (ii) the adventitia is innervated by the sensory and sympathetic NS but not by the parasympathetic NS system; (iii) the adventitia is wired directly to the brain stem through lower thoracic dorsal-root-ganglia and sympathetic perivascular ganglia; (iv) advanced atherosclerosis is associated with increased nerve activities in splenic and celiac vagus nerves; and (v) elimination of the sympathetic NS disrupts ATLOs, attenuates atherosclerosis progression, and reduces plaque vulnerability. Our data demonstrate the pathophysiological relevance of NICIs in atherosclerosis and that the PNS employs NICIs to assemble ABCs. We hypothesize that intervention into neural circuits creates multiple unexpected opportunities to treat atherosclerosis.

## S6. Neural control of immunity in hypertension

### Daniela Carnevale, PhD

#### University di Roma, Rome, IT

The nervous system and the immune system share the common ability to exert gatekeeper roles at the interfaces between internal and external environment. Although interaction between these two evolutionarily highly conserved systems is long recognized, the investigation into pathophysiological mechanisms regulating their reciprocal cross-talk has been the object of pathbreaking immunology and neuroscience research only in recent decades. In the last years, our group elucidated how the autonomic nervous system controls the splenic immunity recruited by hypertensive challenges. In this lecture, I will focus on the molecular mechanisms that regulate the neuro-immune crosstalk in hypertension. I will elaborate on the mechanistic insights into this brain-spleen axis led us uncover a new molecular pathway mediating the neuroimmune interaction established by noradrenergic-mediated release in the spleen of placental growth factor (PlGF), an angiogenic growth factor potentially targetable with pharmacological approaches.

## S7. Personalized bioelectroceutical modulation of carotid sinus nerve: a novel approach to treat type 2 diabetes

### Silvia V. Conde, PhD

#### CEDOC, NOVA Medical School, Faculdade Cinecias Médicas, Universidade NOVA de Lisboa, Lisboa, PT

It is consensual that increased carotid body (CB) activity contributes to the sympathetic overactivation that characterizes essential hypertension, hypertension associated with obstructive sleep apnea and chronic heart failure (1). Additionally, we described that CB dysfunction is in the genesis of metabolic diseases via sympathetic nervous system overactivation (2,3).

The CBs are arterial chemoreceptors that sense changes in blood O2, CO2, and pH levels. Apart from ventilatory control, the CBs have been reported to act as metabolic sensors being implicated in the control of energy homeostasis. We have recently described that CB activity is increased in animal models of metabolic disease (2,4) and in prediabetes patients (5). Also, we showed that abolishment of CB activity via bilateral resection of CB-sensitive nerve, the carotid sinus nerve (CSN), or via CB ablation in animals prevents and reverses diet-induced insulin resistance and glucose intolerance as well as sympathoadrenal overactivity, meaning that the beneficial effects of decreasing CB activity on glucose homeostasis are modulated by target-related efferent sympathetic nerves, through a reflex initiated in the CBs (2,3,6). In agreement with our pre-clinical data, we described that hyperbaric oxygen therapy, that dramatically reduces CB activity, improves glucose homeostasis in type 2 diabetes (T2D) patients (7). Unilateral and/or bilateral CB surgical ablation has been also proposed as therapeutic option for sympathetic-mediated diseases. However, despite the promising results, neither hyperbaric oxygen therapy nor surgical CSN resection are optimal therapeutic approaches for CB-mediated diseases, as these procedures entail several side effects. My team recently showed that electrical modulation of the CSN restores glucose homeostasis in T2D animals implying that bioelectronic medicines might be suitable and selective therapeutic strategy for metabolic diseases. The present talk will provide a state-of-the-art update on the mechanisms of sensory transduction, neural circuitry, and reflex regulation of CBs chemoreceptor in metabolic diseases and will discuss the recent findings showing the efficacy of continuous kilohertz frequency alternative current device implanted on the CSN to reverse clinical features of T2D in rats (6) as well as the use of CSN neural recordings to detect metabolic information (8) that could be used for closed-loop control of CSN neuromodulation.

1. Iturriaga R. 2018 J Physiol. 596:3067-3077

2. Ribeiro MJ et al. 2013 Diabetes. 62:2905-16

3. Sacramento JF et al. 2017 Diabetologia. 60:158-168

4. Dos Santos E et al. 2018 Adv Exp Med Biol 1071:103-108

5. Cunha-Guimaraes JP et al. 2020 Eur J Endocrinol. 182:549-557

6. Sacramento JF et al. 2018 Diabetologia 61:700-710

7. Vera-Cruz P et al. 2015 Adv Exp Med Biol. 860:221-5

8.Cracchiolo M et al. 2019 IEEE Trans Neural Syst Rehabil Eng. 27:2034-2043

Supported by: Portuguese Foundation for Science and Technology (PTDC/SAU-ORG/111417/2009/ SFR/BD/88983/2012; PD/BD/105890/2014), L’Oreal/FCT/Unesco Medals

## S8. AI and bioelectronic medicine: A vision for future research and collaboration

### Suraj Kapa, MD

#### Mayo Clinic, Rochester, MN, US

Artificial intelligence and machine learning are methods by which a computer system can generate complex, non-linear algorithms from data inputs that have the potential to be more accurate and deployed in real-time. In bioelectronics medicine, there are multiple pieces of complex electrophysiologic data that are acquired. While traditional diagnostic and therapeutic responses from these data are, by necessity, based on relatively simple models, the use of artificial intelligence may allow for the creation of more dynamic and complex responses that better mirror how normal human physiology responds to these electrical impulses. In the field of cardiac electrophysiology, for example, it has been demonstrated that, using a standard 12-lead electrocardiogram, it is possible to detect several diseases that are not apparent according to traditional interpretation methods, such as the risk of having a low ejection fraction, atrial fibrillation, or hypertrophic cardiomyopathy. In turn, dynamic, closed loop feedback systems that integrate several pieces of electrophysiologic data and determine appropriate responses that mirror physiologic needs may be feasible. Future research will focus on how bioelectronics data may be aggregated, used to build predictive algorithms, and how these algorithms may be integrated into diagnostic and therapeutic systems to advance bioelectronic devices.

## S9. Organic bioelectronics operating at the speed and resolution of neuronal signalling

### Magnus Berggren, PhD

#### Department of Science & Technology, Laboratory of Organic Electronics, Linköping University, SE

Organic electronic materials are unique as the signal translator across the biology-technology gap. These biocompatible materials are also easily complexed with polyanions, polycations and functional biomaterials and can be included in various device architectures to form flexible, stretchable and even gelled device systems. Such organic bioelectronics can then process electronic, ionic and charged biomolecules in combination. These combined features make organic electronic materials unique in many aspects as the recorder and actuator of various functions and physiology of biological systems.

A brief review of some of the recent achievements from the Laboratory of Organic Electronics is here given. In the BioComLab technology platform various organic bioelectronic sensors and actuators are combined with communication technology to form a body area network for future healthcare applications. Various sensors are included within electronic skin patches, then connected to electronic drug delivery components via capacitive body-coupled communication. This system provides sensor-actuator feedback and improves its decision-making performance using deep-learning protocols provided from cloud connectivity. With the BioComLab platform we target an array of neuronal disorders and diseases, such as epilepsy, Parkinson’s disease and chronical pain.

## S10. Bioelectronics in Gastrointestinal Sphincters

### Larry Miller, MD, Anil Vegesna, MD, Joel Zachariah, BS, Jason Wright, MEng, Nikunj Bhagat, PhD, Loren Rieth, PhD^,^ Timir D. Chaudhury, PhD and Stavros Zanos, MD PhD

#### Institute of Bioelectronic Medicine, Feinstein Institutes for Medical Research, Manhasset, New York, US

##### Larry Miller

A sphincter is a ring like muscle that normally maintains constriction of a body passage or orifice and that relaxes as required by normal physiological functioning. Sphincters are control mechanisms in the gastrointestinal tract. They control forward passage and prevent or allow the backwards passage of solids, liquids and gases. Direct electrical stimulation of the sphincter can lead to contraction while stimulation of the Vagus Nerve can lead to contraction or relaxation of the sphincter, depending on the parameters of stimulation.

Currently most vagal nerve stimulation is performed at the level of the Cervical Vagus Nerve. This stimulation leads to specific targeted physiologic effects at the expense of other untargeted physiologic effects. We propose stimulating peripheral branches of the vagus nerve near the target organ to more precisely target desired specific physiologic effects without the unwanted physiologic effects.

We have developed tunneling techniques in a porcine model in which a Z tract is created within the submucosal space of the esophagus, stomach, small intestine or colon. These techniques can be used to access peripheral branches of the Vagus Nerve in order to place electrodes and more precisely target beneficial effects of nerve stimulation while eliminating unwanted effects. Through the esophagus one can reach vagal branches to lungs, heart, esophagus, and stomach. Through the stomach one can reach vagal branches to stomach, liver, pancreas, kidneys, small intestine, spleen and colon.

We have also developed the use of endoscopic ultrasound, in a porcine model, to direct needle electrodes to target, access and stimulate nerves and ganglion within the Celiac, Superior Mesenteric and inferior Mesenteric Plexus.

Using FLIP technology to evaluate the contraction and relaxation of the pyloric sphincter and the lower esophageal sphincter we have demonstrated both contraction and relaxation of these sphincters using direct electrical stimulation of muscle and indirect electrical stimulation of the vagus nerve.

We believe that these techniques may be used in the future to access and stimulate peripheral branches of the Vagus Nerve to treat many diseases including, chronic hypertension, diabetes, gastroesophageal reflux disease (GERD), gastroparesis, end stage congestive heart failure (CHF), end stage pulmonary disease such as pulmonary hypertension, asthma, diabetes, renal failure, IBD, other inflammatory diseases and obesity, as well as diseases related to dysfunction of gastrointestinal sphincters.

## S11. Visual cortical prosthetics: The next generation

### Daniel Yoshor, MD

#### University of Pennsylvania, Philadelphia, PA, US

This abstract from this presentation is adapted from the following publication: Beauchamp MS, Oswalt D, Sun P, Foster BL, Magnotti JF, Niketeghad S, Pouratian N, Bosking WH, Yoshor D. Dynamic Stimulation of Visual Cortex Produces Form Vision in Sighted and Blind Humans. Cell. 2020;181(4):774-83 e5. Epub 2020/05/16. doi: 10.1016/j.cell.2020.04.033. PubMed PMID: 32413298; PMCID: PMC7331799.

A visual cortical prosthesis (VCP) has long been proposed as a strategy for restoring useful vision to the blind, under the assumption that visual percepts of small spots of light produced with electrical stimulation of visual cortex (phosphenes) will combine into coherent percepts of visual forms, like pixels on a video screen. In prior decades, a number of research teams tested early prototypes of a VCP, but these efforts were limited by the technology of the day. The remarkable advances in computer science and engineering have spurred a renewed interest in developing VCPs that is now well underway. In this presentation I review past and ongoing work in developing VCPs. In addition, I highlight the challenges involved in using phosphenes as combinatorial elements, and describe an alternative strategy in which shapes are traced on the surface of visual cortex by stimulating electrodes in dynamic sequence. In our experience testing both sighted and blind participants, dynamic stimulation enabled accurate recognition of letter shapes predicted by the brain's spatial map of the visual world. Forms were presented and recognized rapidly by blind participants, up to 86 forms per minute. These findings demonstrate that a brain prosthetic can produce coherent percepts of visual forms.

## S12. Keynote Talk: Modulation of the α7 nicotinic acetylcholine receptor (α7nAChR) in the anti-inflammatory function of the vagus nerve

### Lawrence Steinman, MD

#### Stanford University School of Medicine, Stanford, CA, US

The abstract from this presentation is adapted from the following publication: Rothbard JB, Kurnellas MP, Ousman SS, Brownell S, Rothbard JJ, Steinman L. Small Heat Shock Proteins, Amyloid Fibrils, and Nicotine Stimulate a Common Immune Suppressive Pathway with Implications for Future Therapies. Cold Spring Harb Perspect Med. 2019 Jul 1;9(7):a034223. doi: 10.1101/cshperspect.a034223. PMID: 30249602; PMCID: PMC6601455.

The α7 nicotinic acetylcholine receptor (α7nAChR) is central to the anti-inflammatory function of the vagus nerve in a physiological mechanism termed the inflammatory reflex. Studies on the inflammatory reflex have been instrumental for the current development of the field of bioelectronic medicine. An independent investigation of the biological role of αB-crystallin (HspB5), the most abundant gene transcript present in active multiple sclerosis lesions in human brains, also led to α7nAChR. Induction of experimental autoimmune encephalomyelitis (EAE) in HspB5−/− mice results in greater paralytic signs, increased levels of proinflammatory cytokines, and T-lymphocyte activation relative to wild-type animals. Administration of HspB5 was therapeutic in animal models of multiple sclerosis, retinal and cardiac ischemia, and stroke. Structure–activity studies established that residues 73–92 were as potent as the parent protein, but only when it formed amyloid fibrils. Amyloid fibrils and small heat shock proteins (sHsps) selectively bound α7nAChR on peritoneal macrophages (MΦs) and B lymphocytes, converting the MΦs to an immune suppressive phenotype and mobilizing the migration of both cell types from the peritoneum to secondary lymph organs. Finally, our approach in translation to the clinic is for the development of small molecules that are orally active to modulate the α7nAChR.

### **Other speaker talks**

## S13. SPARC, Data and tools from the NIH SPARC program to catalyze the development of next-generation therapeutics

### Gene Civillico, PhD

#### National Institutes of Health

## S14. Designing new materials for Bioelectronics applications

### Molly M. Stevens, PhD

#### Imperial College London

## S15. Non-invasive neuromodulation using ultrasound: A work in translation

### Chris Puleo, PhD

#### General Electric

## S16. Technologies and therapies to enable persons with neurological injuries to reach their full potential

### Robert Rennaker, PhD

#### University of Texas at Dallas

## S17. Neuroimmune cardiovascular interfaces form atherosclerosis brain circuits

### Saroj Mohanta, PhD

#### Institute for Cardiovascular Prevention, Ludwig-Maximilians-University

## S18. Vagus nerve stimulation in Crohn's disease: A 12-month pilot study outcomes

### Bruno Bonaz, MD-PhD

#### CHU and Grenoble Institute of Neurosciences (GIN)-INSERM

## S19. Electrical activation of neural reflexes for the treatment of autoimmune diseases

### David Chernoff, MD

#### SetPoint Medical

## S20. The jump from animals to humans: How to make the jump easier

### Kip Ludwig, PhD

#### University of Wisconsin

## S21. Bioelectronic medicines that look good ON you

### Doug Weber, PhD

#### University of Pittsburgh

