# Correction to: Electrically-evoked vagus nerve recordings using transmural endoscopy in a swine model

**DOI:** 10.1186/s42234-021-00070-y

**Published:** 2021-11-23

**Correction to: Bioelectronic Medicine 6 (2020)**

**https://doi.org/10.1186/s42234-020-00060-6**

Nikunj Bhagat, PhD^1^, Anil Vegesna, MD^1^, Chunyan Li, PhD^1,2^, Loren Rieth, PhD^1,2^, Richard Ramdeo^1^, Chad Bouton, MSc^1,2^, Horacio Rilo, MD^2,3^, and Larry Miller, MD^1,3*^

* Correspondence: Larry Miller

^1^Institute of Bioelectronic Medicine, Feinstein Institutes for Medical Research, Northwell Health, Manhasset, New York, USA

^2^Zucker School of Medicine at Hofstra/Northwell, Hempstead, New York, USA

^3^North Shore University Hospital-Northwell Health, Manhasset, New York, USA

After publication of this supplement (Bhagat et al., 2020), it was brought to the authors’ attention that Figure 1 was mistakenly published in abstract P2. The figure has been removed from the original article.

The correct abstract without Figure 1 is given below:

Transmural endoscopy for the purposes of performing per oral endoscopic myotomy (POEM) is a well-established and routine procedure for treating achalasia. During this procedure, an endoscopic tunnel is formed between the esophageal mucosa to the outer muscle layers, thereby gaining access to thoracic and abdominal cavities. Previously, we have shown that it is possible to identify and localize peripheral branches of the vagus nerve using commercially available endoscopes with digital/optical magnification and narrow band imaging. In this study, we explore the feasibility of recording nerve activity from the esophageal and gastric vagus branches within the abdominal and thoracic cavity in a swine model, by using transmural endoscopy. We will use custom-built nerve cuff and spiral electrodes that can be placed endoscopically to monitor for nerve activity and stimulate at different sites along the peripheral vagal branches. Additionally, we will also record the evoked compound action potentials (CAPs) in response to cervical and auricular vagus nerve stimulation applied directly on the nerve.

Transmural endoscopy opens up the possibility of localizing and recording from the vagal nerve branches via a minimally invasive surgical procedure. Further studies are needed to explore its potential for delivering bioelectronic medicine.

The original article (Bhagat et al., 2020) has been corrected.
